# Clinical diagnosis, treatment and prognosis analysis of children with pancreatic tumours

**DOI:** 10.3389/fped.2025.1576273

**Published:** 2025-06-12

**Authors:** Yao Zhang, Chao Yang, Zhenzhen Zhao

**Affiliations:** ^1^ Department of Surgical Oncology, Children’s Hospital of Chongqing Medical University, National Clinical Research Center for Child Health and Disorders, Chongqing, China; ^2^ Ministry of Education Key Laboratory of Child Development and Disorders, Chongqing, China; ^3^ International Science and Technology Cooperation Base of Child Development and Critical Disorders, Chongqing, China; ^4^ Chongqing Municipal Health Commission Key Laboratory of Children’s Vital Organ Development and Diseases, Chongqing, China

**Keywords:** pancreatic tumour, children, treatment, prognosis, diagnosis

## Abstract

**Objective:**

To investigate the diagnosis, treatment, and prognosis of children with pancreatic tumours.

**Methods:**

We conducted a retrospective review of patients ≤18 years of age with pancreatic neoplasms who underwent surgery at a single institution between 2010 and 2022.

**Results:**

The most common histology among the 48 patients was solid pseudopapillary neoplasm of the pancreas (SPN, *n* = 32), followed by pancreatic blastoma (PB, *n* = 12), B-cell lymphoblastic lymphoma (*n* = 1), leukaemia (*n* = 1), pancreatic cancer (*n* = 1), and pancreatic neuroendocrine tumours (*n* = 1). The tumours were located in the head of the pancreas in 29 patients (10 patients underwent pancreaticoduodenectomy, 9 patients underwent Whipple, 7 patients underwent pancreatic tumour resection, and 3 patients underwent laparoscopic tumour enucleation). The tumours were located in the tail of the pancreatic body in 19 patients (10 patients underwent local resection of the pancreatic tumour, 5 patients underwent resection of the pancreatic body and tail and spleen, 3 patients underwent spleen-preserving distal pancreatectomy, and 1 patient underwent laparoscopic resection of the tumour). Patients with pancreatoblastoma, acute lymphoblastic leukaemia, B lymphoblastic lymphoma or pancreatic cancer were treated with regular chemotherapy. Forty-five patients (93.75%) were currently alive and disease free, and the median follow-up was 8.2 years (IQR: 2.0–14.3). Two patients with pancreatic blastoma died during the follow-up, and 1 patient with pancreatic cancer died due to tumour recurrence and progression after treatment.

**Conclusion:**

Paediatric pancreatic tumours are highly heterogeneous. Complete surgical resection that preserves organ function is the preferred treatment for children with pancreatic tumours.

## 1 Introduction

The incidence of primary pancreatic tumours in children is rare, and according to the National Cancer Institute Surveillance Epidemiology and End Results (SEER-17), the reported incidence of malignant pancreatic tumours in children is only 0.02 per 100,000 children per year. It accounts for 0.2% of all childhood tumours (1). Solid pseudopapillary neoplasm of the pancreas (SPN) and pancreatoblastoma (PB) are commonly observed clinically (2). Other rare types include neuroendocrine (NET), epithelial and nonepithelial/unknown types, such as sarcoma, lymphoma, haemangioma, rhabdomyosarcoma, inflammatory myofibroblastoma (IMT), PNET, and neuroblastoma (3). The treatment methods and survival rates of children with different pathological types of tumours vary. Surgical resection is the most important treatment method for children with primary pancreatic tumours and can improve patient prognosis (4).

SPN is a rare pancreatic tumour also known as Frantz tumour, with an incidence of approximately 0.01 per 100,000 children per year (5). It is a benign tumour derived from exocrine cells, accounting for 0.13%–2.7% of primary pancreatic tumours and fewer than 2% of all exocrine pancreatic tumours (6).

PB is an epithelial tumour derived from acinar cells that is almost exclusively found in children, accounting for approximately 16% of paediatric pancreatic tumours, and is a common malignant pancreatic tumour in children under 10 years of age (7).

Acinar cell carcinoma (ACC) accounts for approximately 1%–2% of adult exocrine pancreatic tumours, with a typical peak age of onset in the late 60 s. However, in children, ACC accounts for less than 6% of all pancreatic cancer cases in the population; the age of onset is 8–15 years, and the male to female ratio is 2.3:1 (8).

Islet cell tumours, also known as pancreatic neuroendocrine tumours (PNETs), are rare tumours that account for less than 5% of all pancreatic tumours, with a current incidence of 0.43 per 100,000 person-years (9). The clinical manifestations of PNETs are generally divided into two main categories: functional and nonfunctional tumours. Functional tumours exhibit definite clinical symptoms secondary to hormone hypersecretion. Functional PNETs include insulinomas, gastrinomas, glucagonomas, VIP omas, and somatostatin tumours. Nonfunctional tumours, which account for 60%–90% of all PNETs, do not exhibit symptoms associated with excessive hormone production (9). In a few cases, PNETs appear in the context of known genetic syndromes such as multiple type I endocrine tumours or von Hippel–Lindau syndrome (10).

Primary pancreatic lymphoma is a very rare type of tumour, accounting for 1%–2% of all extranodal lymphomas (11). It accounts for approximately 5% of all malignant pancreatic tumours, mostly in male patients. Diffuse large B-cell lymphoma is the most common pathological type, accounting for 80% of cases, whereas other types are rare (12).

Due to the rarity and diversity of pancreatic tumours in children, coupled with the lack of standardized treatment regimens, significant challenges have been brought to clinical research and treatment. At present, to the best of our knowledge, few large-scale cohort studies have explored the epidemiology and treatment outcomes of these tumour patients (13). Therefore, this study utilized data from the population-based database of our institution, aiming to more thoroughly identify and evaluate the diagnosis, treatment and prognosis of children with pancreatic tumours.

This study retrospectively analysed the clinical data of 48 children with pancreatic tumours admitted to the Children's Hospital Affiliated with Chongqing Medical University, including five different types of pancreatic tumours, to summarize the diagnosis and treatment experience and surgical strategies for treating children with different pancreatic tumours to provide a reference for the clinical diagnosis and treatment of paediatric patients with pancreatic tumours.

## 2 Data and methods

### 2.1 Individual inclusion criteria

First, the study was approved by the Institutional Review Committee of the Children's Hospital Affiliated with Chongqing Medical University (No. 63, 2025). In accordance with ethical requirements, informed consent was waived in this study.

Children with pancreatic tumours admitted to the Children's Hospital of Chongqing Medical University between January 2010 and December 2022 were included in this study. The inclusion criteria were as follows: (1) had pancreatic tumours, (2) underwent surgical treatment, and (3) were under the age of 18. The exclusion criteria were as follows: (1) no regular postoperative follow-up, (2) incomplete data, and (3) other serious heart diseases, autoimmune diseases, organ failure, etc.

### 2.2 Treatment methods

All the children underwent surgical treatment. Patients who could not be surgically resected were administered neoadjuvant chemotherapy after a definite diagnosis of PB by biopsy and then underwent complete surgical resection after tumour shrinkage. Children with PB require systemic chemotherapy. Among the 12 children with PB in this group, 11 patients received systematic chemotherapy. One patient relapsed after the operation because the family refused to receive chemotherapy. No special treatment is required after SPN or insulinoma surgery in children. For children with pancreatic leukaemia/lymphoma and pancreatic cancer, conventional chemotherapy is given after surgery.

### 2.3 Observation indicators

Clinical data such as age, sex, pathological data, surgical method, chemotherapy, tumour location, serum alpha-fetoprotein and serum amylase levels before and after surgery, distant metastasis and local invasion, and treatment outcomes were collected. Follow-up was conducted via outpatient review and telephone return visits, and relevant imaging examinations, including abdominal ultrasound, CT, MRI, and positron emission tomography, were performed regularly. Serum alpha-fetoprotein (AFP) levels were also measured in children with PBL. Follow-up was performed until December 30, 2024.

### 2.4 Statistical analysis

The Shapiro–Wilk test was used to check normality, measurement data are expressed as the means and standard deviations (*X* ± *S*), count data are expressed as percentages and were compared via the chi-square test or Fisher's exact test, and the LSD test was used for comparisons between two groups. The measurement data were not normally distributed. The Kruskal–Wallis test was adopted, followed by the Dunn method, with a two-tailed test (*P* < 0.05), and the difference was considered statistically significant. All statistical analyses were performed using SPSS software (version 22.0, China).

## 3 Results

### 3.1 General clinical data

Among the 48 children included, 26 had clinical manifestations of abdominal pain, 5 had abdominal pain accompanied by skin jaundice, 13 had an abdominal mass, 1 had diarrhoea accompanied by vomiting, 1 had fever, 1 had episodic hypoglycaemia, and 1 had no obvious symptoms. Postoperative pathology revealed 32 cases of SPN, 12 cases of PBL, one case of B-cell lymphoblastic lymphoma, one case of B-lymphoblastic leukaemia, one case of insulinoma, and one case of pancreatic acinar cell carcinoma. There were 32 cases of SPN, among which 24 were solid cases, 8 were cystic cases, 27 had intact capsules and 5 had incomplete capsules. There were 12 cases of solid PB: 9 cases with intact capsules and 3 cases with incomplete capsules. The capsules of patients with B-lymphoblastic lymphoma and leukaemia were incomplete. One case of insulinoma was accompanied by a complete capsule. Further details are provided in Table 1.

**Table 1:** Demographic characteristics in 48 patients who underwent resection of pancreas.

Characteristic	Frequency, *n* (%)
Age	
≥6 y	38 (79.17)
<6 y	10 (20.83)
Gender	
Female	31 (64.58)
Male	17 (35.42)
Symptoms	
Abdominal, pain	26 (54.17)
Palpation of intraabdominal mass	13 (27.08)
Nausea and vomiting	1 (2.08)
Jaundice	5 (10.42)
Fever	1 (2.08)
Asymptomatic	1 (2.08)
Episodic hypoglycemia	1 (2.08)
Capsular	
Integrity	39 (81.25)
Invasion	9 (18.75)
Tumor size	
≥5 cm	41 (85.42)
<5 cm	7 (14.58)
Tumor type	
SPTP	32 (66.67)
PBL	12 (25)
Lymphoma/Leukemia	2 (4.17)
Insulinoma	1 (2.08)
Pancreatic cancer	1 (2.08)
Tumor location	
Head	29 (60.42)
Body and tail	19 (39.58)

### 3.2 Laboratory and imaging tests

Among the 48 patients admitted to the hospital for blood examination, 9 patients with pancreatoblastoma had elevated serum AFP, and 6 patients had elevated serum direct bilirubin, ranging from 70.2 to 145.6 µmol/L. In nine patients with elevated serum AFP before surgery, AFP was reexamined after surgery and at every hospitalization, and the levels in all patients decreased to the normal range after several postoperative chemotherapies. Abdominal ultrasonography, enhanced CT, and/or MR were performed in all patients, and solid cystic or solid lesions were found in all patients. The tumours were located in the head of the pancreas in 29 patients (3 with common bile duct invasion, 3 with common bile duct and portal vein invasion, 2 with pancreatic duct and duodenal invasion, and 1 with liver and retroperitoneal lymph node metastasis). The tumours were located in the tail of the pancreatic body in 19 patients (tumour enveloped the splenic arteries and veins with omental invasion in one patient and metastasis to both lungs, left supraclavicular fossa lymph nodes, and retroperitoneal lymph nodes in one patient). The tumour was 3–20 cm long.

### 3.3. Surgical methods and treatment

All the children underwent laparotomy under tracheal intubation and general anaesthesia, and operations were successful. The tumours were located in the head of the pancreas in 29 patients (10 underwent duodenum-reserved pancreatectomy, including 5 pancreaticoduodenectomies with the duodenum preserved and 5 pancreaticoduodenectomies with the pylorus preserved), 9 underwent Whipple, 7 underwent pancreatic tumour resection, and 3 underwent laparoscopic tumour resection). The tumours were located in the tail of the pancreatic body in 19 patients (10 patients underwent local resection of the pancreatic tumour, 5 patients underwent resection of the pancreatic body and tail and splenectomy, 3 patients underwent splenic resection, and 1 patient underwent laparoscopic resection of the tumour). Due to the presence of large tumours (one of which was accompanied by metastasis to both lungs, the left supraclavicular fossa lymph node, and the retroperitoneal lymph node), three patients were confirmed as having PB by biopsy, and CT examination after neoadjuvant chemotherapy revealed that the tumours were significantly smaller than before, that the lung lesions disappeared in patients with lung metastasis, and that the tumours were completely removed after surgery. Postoperative pathology revealed PB in all 12 patients. Eleven patients received systemic chemotherapy after surgery, and 4–8 courses of chemotherapy were administered according to the patient's condition after surgery. The chemotherapy regimens for pancreatoblastoma still lack large-sample evidence-based support, and both the regimens and dosages are not uniform. At present, the European Collaborative Research Group on Rare Childhood Tumours (EXPeRT) and most medical centres recommend the PLADO regimen as the first-line chemotherapy regimen for hepatoblastoma (5, 14–16). The recommended dose is as follows: 80 mg/m^2^ cisplatin via intravenous drip for 24 consecutive hours on the first day; 30 mg/m^2^ doxorubicin via intravenous drip for 24 h on the second and third days; and every 21 days constituting one chemotherapy cycle. According to the stage of the tumour, the following chemotherapy courses are recommended: (1) Stage I, for children with complete tumour resection, 4 courses of adjuvant chemotherapy are administered after the operation; (2) In stage II, children who receive resection with residual or lymph node involvement under the microscope receive 6 courses of adjuvant chemotherapy; (3) For children in stages III and IV who have undergone partial resection or simple biopsy or have tumour rupture and distant metastasis before or during the operation, 4–6 courses of treatment before the operation and more than 2 courses after the operation are recommended. In clinical practice, the specific number of treatment courses varies and is often determined based on the actual plan, combined with the decline in AFP and the therapeutic effect. If the PLADO regimen is ineffective or if the chemotherapy regimen needs to be changed due to the cumulative dose limit of doxorubicin, the ICE regimen (ifosfamide + carboplatin + etoposide) is recommended. The specific dose can refer to the CCCG-HD-2016 regimen (17), the malignant germinal cell tumour regimen, etc. One child refused chemotherapy, and one child underwent salvage surgery and chemotherapy after recurrence, as shown in Table 2.

**Table 2:** Disease staging and treatment modalites.

Variables	Total (*n* = 48)	SPN (*n* = 32)	PB (*n* = 12)	Lymphoma/Leukemia (*n* = 2)	Insulinoma (*n* = 1)	Pancreatic cancer (*n*= 1)
Sex						
Male	17	6	7	2	1	1
Female	31	26	5	–	–	–
Localization						
Head	29	22	5	1	–	1
Body	8	4	3	0	1	–
Tail	7	5	2	–	–	–
Overlapping	4	1	2	1	–	–
Stage						
Localized	32	29	2	–	1	–
Regional	11	3	7	1	–	–
Distant	5	–	3	1	–	1
Chemotherapy						
Yes	14	0	11	2	0	1
No	34	32	1	0	1	0
Operation style						
DPPHR	5	3	2	–	–	–
PPPD	5	3	2	–	–	–
Whipple	9	5	3	1	0	0
Distal Pancreatectomy	2	1	1	–	–	–
Tumorectomy	17	15	2	–	–	–
Laparoscopic tumor resection	3	2	1	–	–	–
SPDP	4	2	–	1	1	–
Pancreatic body tail and spleen resection	3	1	1	–	–	1

SPDP, spleen-preserving distal pancreatectomy; DPPHR, Duodenum-preserving pancreatic head resection; PPPD, pylorus-preserving pancreatectomy.

### 3.4 Prognosis and complications

Short-term complications were assessed according to the International Pancreatic Surgery Research Group (ISGPS) criteria (18–21). In this study, there was 1 case of grade A pancreatic fistula, 1 case of grade B pancreatic fistula, 1 case of chylous fistula, 2 cases of infection, and 2 cases of grade B delayed gastric emptying (Table 3). All patients were successfully discharged with conservative treatment. There were no significant differences in operation time, somatostatin application time, or postoperative fasting time between the head of the pancreas and the body and tail of the pancreatic tumour. In terms of blood loss, the head of the pancreas and tail tumours had more bleeding than the body and tail tumours did, and there was a statistically significant difference between the two (*P* < 0.05) (Table 4). The median follow-up time was 82 months (6–174 months).

**Table 3:** Postoperative complications.

Variables	Total (*n* = 48)	SPN (*n* = 32)	PB (*n* = 12)	Lymphoma/Leukemia (*n* = 2)	Insulinoma (*n* = 1)	Pancreatic cancer (*n* = 1)
Pancreatic Fistula	2	1	1	–	–	–
Infection	2	1	1	–	–	–
Chylothorax	1	–	1	–	–	–
Gastric retention	2	1	1	–	–	–
Chronic pancreatitis	1	–	1	–	–	–
Endocrine insufficiency	1	–	–	–	1	–

**Table 4:** Comparison of the short-term complications between the different primary tumor sites.

Variables	Primary tumor site		Results	*P*
	Pancreatic head	Pancreatic body and tail		
Number of patients	29	19		
Operative time (median, min)	75 (10–800)	100 (30–300)	0.82	0.41
Blood loss (median, ml)	270 (50–420)	150 (100–300)	2.11	0.035
Somatostatin (*n*, %)	5 (0–7)	3 (0–8)	1.95	0.05
Days until starting liquid diet (median, day)	5 (1–21)	6 (1–15)	1.05	0.29

Among the 48 patients, 45 survived without disease, and 3 (6.25%) died. One patient with pancreatoblastoma died of tumour recurrence with multiple metastases, and one patient with pancreatoblastoma died of liver metastasis. One patient with pancreatic cancer died of tumour recurrence with multiple metastases throughout the body. Only two patients experienced long-term complications: one had chronic pancreatitis, and one had endocrine insufficiency (Table 3).

## 4 Discussion

Paediatric pancreatic tumours are rare and have a good prognosis. Its clinical manifestations lack specificity and include mostly abdominal pain, an abdominal mass, and jaundice. Abnormal hormone secretion can lead to specific clinical symptoms in functional endocrine tumours of the pancreas.

Our study provides a retrospective analysis of five types of pancreatic tumours in children from a single institution from 2010 to 2022. The most common histological type was solid pseudopapillary tumours, which have the best prognosis. The overall treatment strategy for pancreatic tumours is surgery. After the operation, chemotherapy is supplemented according to the patient's condition, and radiotherapy is less commonly used.

SPN is a common pathological type of paediatric pancreatic tumour, accounting for more than 50% of paediatric pancreatic tumours, mainly in teenage girls (22).

In adults, the tail of the pancreatic body is the most common site, whereas in children, the head of the pancreas is the most common site, which is consistent with the results of this study. SPN imaging findings revealed the following: (1) well-bounded round or quasi-round solid or cystic lesions of the pancreas; (2) on a plain CT scan, the lump has an uneven cystic and solid area, and the solid part of the CT can be enhanced; and (3) the head of the pancreas can compress the bile duct and pancreatic duct, and magnetic resonance cholangiopancreatography (MRCP) revealed dilation of the bile duct and pancreatic duct. (4) Lymph node and distant metastases are rare. In this group of 32 patients with SPN, the male-to-female ratio was 3:13, and most patients were female. All the children were healthy and alive after 6–174 months of follow-up, which was consistent with relevant foreign reports. Muhammed Ali Colak (3) reported that the 5-year survival rate for patients with SPN was nearly 100%. Preoperative examination revealed that the pancreas had clear boundary cystic or solid lesions, large lesions, a tumour length of 4–12 cm and no invasive manifestations. The treatment for this tumour is surgical resection without the need for chemoradiotherapy.

The 5-year survival rate for patients with pancreatoblastoma is approximately 66.7% (3). The 5-year survival rate for the children in this study was approximately 83.3%, which was related to the treatment differences among different study centres and the small number of cases in this study. The average age of PB onset is approximately 4 years, there are more males than females (the ratio of males to females is approximately 2:1), AFP can be elevated, and most cases are located in the head of the pancreas (7, 23). In this group of 12 children with PB, the youngest age was 3 months, the oldest was 13 years, and the male-to-female ratio was 7:5, which was consistent with relevant reports at home and abroad. PB has no specific serological tumour marker, and it has been reported that 25%–78% of patients with PB have elevated serum AFP (24). In this group, the AFP level increased in seven patients, accounting for 58.3% (7/12) of patients, and decreased to normal after tumour resection, suggesting that the serum AFP level can be used as an indicator to evaluate the efficacy of PB and monitor recurrence. The imaging findings of PB were as follows: (1) the tumour was large in size and prone to cystic degeneration and necrosis; (2) calcification in the tumour was common on plain CT scan, the tumour showed uneven enhancement after CT enhancement, and small blood vessels were visible in the internal or peripheral areas. (3) Easy invasion of the surrounding organs or blood vessels, blood metastasis, or lymphatic metastasis. Among the 12 children in this group, the tumours were large in volume and 4–20 cm in diameter. The intraoperative tumours were all solid, complete in nine patients (R0), incomplete in three patients (R1/2), located in the head of the pancreas in six patients, located in the tail of the pancreatic body in six patients, distant metastasis occurred in 33.33% (4/12), and the metastatic parts were the lungs and lymph nodes.

With a low risk of malignancy and few distant metastases, its treatment is mainly surgical resection and postoperative adjuvant chemotherapy, mainly a cisplatin/adriamycin regimen (7). Although adjuvant radiotherapy has shown promising results, no standardized treatment regimen has been established, and the long-term prognosis is good. The importance of AFP monitoring in treatment planning and recurrence monitoring of pancreatic blastoma.

Pancreatic cancer is a malignancy with a poor prognosis and high mortality, with a crude mortality of 5.7/10^5^ (25). The 5-year survival rate is 10% (26). In this study, the postoperative survival time of the child was 11 months. Other studies reported that the median time was 12 months, and the head of the pancreas was the most common site. The first treatment was surgical resection, followed by chemoradiotherapy and immunotargeted therapy. Early diagnosis and timely surgical intervention are the only effective means to improve the prognosis of pancreatic cancer patients.

Surgery remains the core of treatment; PNETs located at the tail of the pancreas are usually treated with distal pancreatectomy, whereas lesions on the head of the pancreas are usually treated with tumour resection and removal of adjacent lymph nodes or even pancreaticoduodenectomy if the pancreatic tumour is large. Finally, patients with diffuse islet cell carcinoma without distant metastasis require total pancreatectomy (27, 28). In recent years, laparoscopic pancreatectomy has been shown to be a viable option for open surgery in adults. However, given the limited evidence supporting the accuracy of preoperative localization studies in children and the critical role of intraoperative palpation and intraoperative ultrasound in identifying PNETs in children, exploratory laparotomy rather than laparoscopy is still recommended in children (29).

Primary pancreatic lymphoma is most common in the fifth and 60th years of life. In this study, the tumours in two children were located in the head of the pancreas, and the disease could not be identified before surgery. Whipple surgery was performed in both patients without postoperative complications, and they were diagnosed with stage I/II low-risk disease after surgery. The first-line chemotherapy regimen was prednisone-vincristine, adriamycin, and cyclophosphamide, and the patient survived well after surgery. In some CD20-positive diffuse large B-cell cases, the addition of rituximab to the above regimen increased the response rate (12). Symptoms include jaundice, pancreatitis, abdominal pain, abdominal masses, and diarrhoea; however, typical type b symptoms of non-Hodgkin lymphoma include night sweats or fever (30). The median OS for primary pancreatic lymphoma/leukaemia patients is 6.1 years, and the 10-year disease-specific survival is 69% (31).

In conclusion, surgical resection is the best treatment option for children with pancreatic tumours (32, 33). Extensive organ tissue resection and corresponding duct reconstruction may result in the suppression of pancreatic function, even if the tumour is completely removed. Some studies have reported that children with pancreatic tumours who undergo pancreaticoduodenectomy (childhood operation) have developmental delays in height and weight compared with children of the same age after surgery and present moderate and mild malnutrition, which is considered to be related to the removal of too many organs and the destruction of intestinal continuity in children (34). Studies have shown that an insufficient amount of remaining pancreatic tissue after pancreatectomy is related to pancreatic dysfunction, and the degree of impaired glucose metabolism is closely related to the number of parenchymal pancreatectomies (35). In this group, 9 children underwent Whipple surgery, and there was no significant difference in the growth and development of the children compared with children of the same age during postoperative follow-up, indicating that pancreaticoduodenectomy is safe for children with pancreatic tumours. The overall prognosis of children with pancreatic tumours is good. In this study, the incidence of short-term and long-term postoperative complications was 18.75%, which was lower than the 20%–30% reported complications. Surgical strategies and chemotherapy regimens are customized according to tumour subtypes, whereas aggressive surgical procedures, such as Whipple, are safe and effective in paediatric patients. However, the number of patients in this group was small, and this was a single-centre study; thus, the conclusions need further verification. Children are in a critical period of growth and development, and maintaining the continuity of the gastrointestinal tract, the immune function of the spleen, and the internal and external secretion functions of the pancreas are crucial for their growth and development. Therefore, complete resection with functional preservation has become the preferred treatment for children with pancreatic tumours.

Chemotherapy plays an important role in reducing the volume of unresectable PB tumours and preventing postoperative recurrence. Due to the large tumours, the three children in this group were confirmed to have PB by biopsy, and CT examination after neoadjuvant chemotherapy revealed that the tumours were significantly reduced and that the lung lesions disappeared in patients with lung metastasis. Among the 12 pancreatoblastoma patients in this group, 11 received systematic chemotherapy, and all survived after postoperative follow-up without tumour recurrence; one patient refused chemotherapy after surgery, underwent salvage surgery and chemotherapy for tumour recurrence during follow-up, and survived for a long time. For pancreatoblastoma with large volumes and special locations, it is recommended to first reduce the tumour volume and scope of resection with chemotherapy, followed by complete resection with second-stage surgery. The chemotherapy regimens used for pancreatoblastoma differ across medical centres. This centre mainly adopts the PLADO chemotherapy regimen and comprehensively adjusts the chemotherapy regimen in combination with factors such as the AFP level of the child after chemotherapy, the toxicity and side effects of chemotherapy, and the RECIST guidelines (version 1.1) (36). The main drugs used were cisplatin, vincristine, doxorubicin, bleomycin, and etoposide for 4–6 courses of treatment before surgery and for 6–10 courses of treatment after surgery.

Because the pancreatic tumour microenvironment is different from that of other tumours, immunotherapy and tumour targeting have not achieved significant results in pancreatic tumours (37). Chemotherapy is still the main treatment method for pancreatic cancer; however, pancreatic cancer is a refractory tumour with a very poor prognosis. The exploration of drugs, including immune checkpoint inhibitors, tyrosine kinase inhibitors, and the targeting of metabolic pathways or tumour microenvironments is urgently needed (38). Moreover, the search for reliable prognostic biomarkers is critical for optimizing cancer treatment strategies, especially in the era of personalized medicine.

This study is a retrospective case series with a relatively small number of patients and therefore has several limitations. Moreover, the lack of a normal control group may lead to a lack of statistical analysis and meaningful results. In addition, improving overall survival for patients with malignancies and a poor prognosis is a major challenge, and we need to screen for reliable biomarkers in blood, cystic fluid, or pancreatic fluid. For example, circulating tumour DNA, methylation biomarkers, miRNAs and proteomics have been used for early detection and treatment, while other new therapeutic methods, such as targeted therapy based on gene detection and immunotherapy, have been actively explored.

In summary, B-ultrasound, CT, and MRI cannot easily locate and diagnose paediatric pancreatic tumours preoperatively, and MRCP and CT can be used to understand the positional relationships between tumours and the bile duct, pancreatic duct, and peripheral blood vessels, providing a basis for accurate resection of pancreatic tumours. Surgical resection is the most important treatment method for paediatric pancreatic tumours, with functional preservation and complete resection as the criteria for surgical selection. SPN can result in a good prognosis only through complete surgical resection without special treatment. For PB, preoperative neoadjuvant chemotherapy can significantly shrink the tumour and create conditions for complete tumour resection. Through surgery combined with chemotherapy, children achieve long-term survival.

For patients with pancreatic blastoma tumours that are not resectable at diagnosis, preoperative biopsy and preoperative chemotherapy are recommended to reduce the tumour volume, and a second complete resection combined with chemotherapy can still lead to long-term survival. There are differences in the treatment and prognosis of different types of pancreatic tumours.

## 5 Conclusion

Paediatric pancreatic tumours are highly heterogeneous tumour lesions. Complete surgical resection that preserves organ function is the preferred treatment for children with pancreatic tumours.

## Data Availability

The raw data supporting the conclusions of this article will be made available by the authors, without undue reservation.
